# Impact of Coronary Tortuosity on Coronary Blood Supply: A Patient-Specific Study

**DOI:** 10.1371/journal.pone.0064564

**Published:** 2013-05-17

**Authors:** Xinzhou Xie, Yuanyuan Wang, Hongmin Zhu, Hu Zhou, Jingmin Zhou

**Affiliations:** 1 Department of Electronic Engineering, Fudan University, Shanghai, China; 2 Department of Cardiology, Zhongshan Hospital, Fudan University, Shanghai Institute of Cardiovascular Diseases, Shanghai, China; University of Arizona, United States of America

## Abstract

**Background:**

Tortuous coronary arteries are commonly observed in clinical screenings and it may cause a reduction of the coronary pressure. However, whether this reduction leads to significant decreasing in the coronary blood supply is still unknown. The purpose of this study was to investigate the effect of the coronary tortuosity (CT) on the coronary blood supply.

**Method:**

A computational fluid dynamics (CFD) study was conducted to evaluate the impact of tortuosity on the coronary blood supply. Two patient-specific left anterior descending coronary artery (LAD) models and the corresponding non-tortuous models were reconstructed to perform three-dimensional CFD analysis. The lumped parameter model was coupled to the outlet of the simulated branches to represent the absent downstream vasculatures. The rest and exercise conditions were modeled by specifying proper boundary conditions.

**Result:**

Under resting condition, the mean flow rate could be maintained by decreasing less than 8% of the downstream vascular bed's resistance for tortuous models. While during exercise (maximal dilatation condition), the maximal coronary blood supply would reduce up to 14.9% due to tortuosity. Assuming that the flow rate can be maintained by the auto-regulation effect under the maximal dilatation condition, the distal resistances for CT models still have to reduce more than 23% to maintain blood perfusion.

**Conclusions:**

Coronary tortuosity has minor influence on coronary blood supply at rest; while during exercise, patients with CT may lack the ability to adjust distal resistance sufficiently to compensate for the extra resistances generated by tortuosity and this may further lead to an ineffective regulation of the blood supply.

## Introduction

Arteries are normally straight to efficiently transport blood to distal organs. However, arteries may become tortuosity as a result of arterial remodeling [1]. Tortuosity is widely observed in coronary arteries [1]–[3]. However, the etiology and clinical importance of the coronary tortuosity (CT) are still unclear. Clinical observations have linked CT to aging, atherosclerosis, hypertension and diabetes mellitus [1]–[2]. Li *et al.* reported that CT was positively related with essential hypertension and female gender but negatively linked with the coronary atherosclerosis [2]. Recently, more and more clinical findings implied that although mild CT was a common anomaly without clinical symptoms, severe CT may bring about various symptoms. Tortuous coronary arteries may hamper the ventricular function and have been proposed as an indicator of the ventricular dysfunction [4]. CT is associated with reversible myocardial perfusion defects and chronic stable angina [5]–[6]. Patients with CT often suffer the exercise-induced chest pain and typically disappear at rest [3]. These clinical findings imply that CT may hinder coronary blood supply. However, no clinical data can directly support it. Whether coronary tortuosity will lead to a significant decreasing in the coronary blood supply is still unknown.

Computational fluid dynamics (CFD) offers a new way to study this problem. CFD is a branch of fluid mechanics which uses numerical methods and algorithms to solve and analyze problems about fluid flows. By using reconstructed geometries and proper boundary conditions, an accurate reconstruction of the blood flow in physiological and pathological conditions can be obtained. Recently, Li *et al.* studied the impact of tortuosity on coronary pressure by using the CFD method [7]. In their work, they simplified the simulation to a 2D CFD study and idealized geometry models were used. Additionally the coronary flow was prescribed, not predicted in their study. They simulated the blood flow under resting condition and concluded that CT would decrease the perfusion pressure. However, due to the auto-regulation effect, the coronary blood flow may be maintained constant in the face of a change in perfusion pressure [8]. Thus the impact of tortuosity on the coronary blood supply cannot be assessed in their studies. A more comprehensive model should be used to study the impact of tortuosity on coronary blood supply.

As numerical methods have advanced, more realistic boundary conditions have been developed in an effort to consider the interactions between the computational domain and the absent upstream or downstream vasculatures [9]. These boundary conditions represent the upstream and downstream vasculatures using simple models such as resistance, impedance, lumped parameter models and couple to computational models [9]. With this approach, coronary flow can be predicted and the impact of CT on the coronary blood supply can be investigated. By considering a hybrid numerical/analytic closed-loop system, Kim *et al.* developed a method to calculate flow and pressure in three-dimensional coronary vascular beds [10]–[11]. In their work, they solved for coronary flow and pressure as well as aortic flow and pressure in subject-specific models by considering the interactions between the model of the heart, the impedance of the systemic arterial system, the pulmonary system, and the impedance of coronary vascular beds. By specifying proper parameter values for the lumped parameter models, coronary flow during exercise was also investigated. In our work, this simulation model was simplified and then employed to provide a realistic boundary condition.

Coronary arteries deliver oxygen-rich blood to the myocardium. Blocked coronary arteries will lead to ischemia and cause angina or even heart attack. CT may block the coronary flow but little attention was paid on this area until recently. Studies on CT are limited in clinical researches and these clinical researches highlight the need for computational studies on the underlying hemodynamic factors. The main objective of this study was to investigate how the coronary blood supply was affected by tortuosity based on two individual patient cases. In this paper, two patient-specific left anterior descending coronary arteries (LAD) and the corresponding non-tortuous artery models were reconstructed. The lumped parameter coronary vascular model was coupled to the outlet of the three-dimensional model to represent the downstream vascular beds absent in the computational domain. By specifying proper boundary conditions, the rest and exercise conditions were modeled to investigate the impact of tortuosity on the coronary blood supply in different physiological conditions.

## Method

### Ethics Statement

This work is a computational simulation study and the ethical approval was not required.

### Vascular Geometry Reconstruction

**Figure 1:**
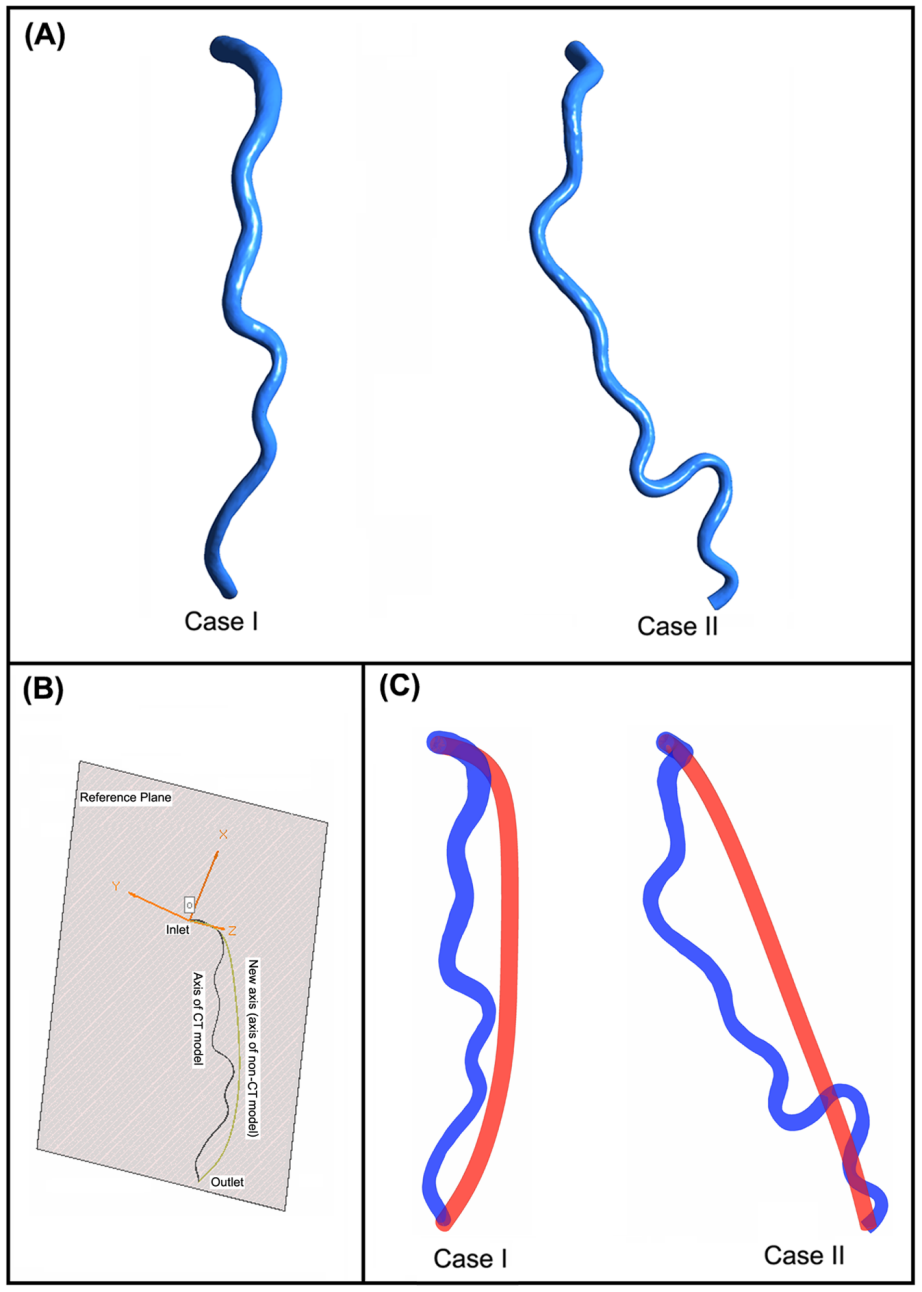
Vascular Geometry Reconstruction. (A) Reconstructed 3D patient-specific LAD models. (B) The construction of the non-CT models (C) The patient-specific LAD models (red) and the corresponding non-tortuous artery models (blue).

The LAD branches of two patients with tortuosity were reconstructed based on multislice computerized tomography images acquired at mid-diastole, 69% into the duration of the cardiac cycle, using SOMATOM Definition (Siemens Medical Systems, Forchheim, Germany) computerized tomography scanner. The slice interval was 0.4 mm and the in-plane image resolution was 0.43 mm/pixel. The LAD branches were semi-automatically segmented from the stack of cross-sectional images. Then the 3D models were reconstructed in Mimics (Mimics, Materialise, Leuven, Belgium). The reconstructed LAD models were shown in Figure 1 (A).

For each LAD model, a non-tortuous model was constructed as “the non-CT LAD branch”, in order to make a comparison between CT models and non-CT models. The axis of non-CT models was constructed based on the axis of the reconstructed patient-special LAD models. First, the inlet of CT models was constrained in the XY plane. Then a reference plane was defined by the z-axis and the end point of the patient-special LAD's axis. Finally the new axis was generated by projecting the axis of the patient-special LAD models onto the reference plane. The construction of the new axis was illustrated in Figure 1 (B). The non-tortuous models were then swept out as 3D pipes along the new axis and the diameter was set as the mean diameter of the reconstructed patient-special LAD models. The two reconstructed geometries and the corresponding non-CT models were depicted in Figure 1 (C). By using this method, the tortuosity was removed from the CT models while the curvature of the CT models was retained. (Coronary arteries are on the surface of the heart and the curvature refers to the curvature of the heart.) In clinical, the level of tortuosity is described by the tortuosity indices. A commonly used tortuosity index is defined as the ratio of vessel curve length over the line distance between the two ends [1]. Alternatively, the tortuosity index can be defined as the cumulative sum of the angle between segment vectors normalized by vessel length [1]. These tortuosity indices indicated that both the “bend effect” and the “lengthen effect” are important factors in characterizing CT. In this work, compared to non-CT models, the tortuous coronary arteries have some bend sections and are longer than non-CT models. Thus, the two important geometry changes caused by tortuosity are both included and the constructed non-CT models were physiological. The mean diameters of the LAD models were: 0.24 cm for Case I (Figure 1 Left) and 0.22 cm for Case II (Figure 1 Right). The curve lengths for four models were: 9.17 cm for Case I CT model, 8.38 cm for Case I non-CT model, 10.85 cm for Case II CT model and 8.58 cm for Case II non-CT model.

### Boundary Conditions

Kim *et al.* developed a hybrid numerical/analytic closed-loop system to calculate flow and pressure in three-dimensional coronary vascular beds [10]–[11]. In this system, a subject-specific model containing aortic and coronary arteries was reconstructed. The aortic inlet was coupled to a lumped parameter model of the left ventricle. The aortic and the coronary outlets were coupled to lumped parameter models and then feed back to the model representing right sides of the heart. Pulmonary circulation was also included in this model and a complete cardiovascular system was built up.

**Figure 2:**
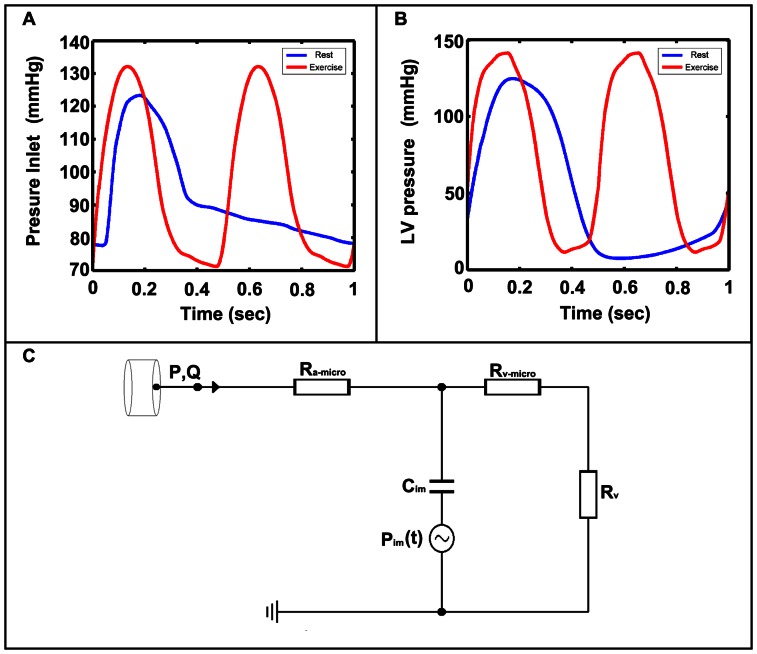
Boundary conditions. (A) The inlet pressure waveform at rest (blue) and during exercise (red). (B) The LV pressure waveform at rest (blue) and during exercise (red). (C) The lumped parameter model.

In our work, we concentrated on the flow of the LAD branch and the hybrid numerical/analytic closed-loop model was simplified to reduce the computing cost. The mean coronary flow was about 3% of the cardiac output [12]. Simulation results have shown that the mean LAD flow was less than 40% of the mean coronary flow [10]. Thus the mean LAD flow was only about 1.2% of the cardiac output. In this work, we assumed that the feedback of the LAD branch (1.2% of the cardiac output) did not affect the heart's lumped parameter model. To simulate the flow of the LAD branch, the closed-loop system can be broken up. In our model, the upstream lumped parameter models were cut off and pressure profiles obtained from Kim *et al.*'s model were directly implied to the inlet of the LAD branch. The outlet of the LAD branch was coupled to a lumped parameter model and was shown in Figure 2. This vascular bed model consists of coronary arterial microcirculation resistance *R_a-micro_*, myocardial compliance *C_im_*, coronary venous microcirculation resistance *R_v-micro_*, coronary venous resistance *R_v_*, and intramyocardial pressure *P_im_*(*t*).

The capacitance *C_im_* mimics the large intramyocardial compliance. The intramyocardial pressure *P_im_*(*t*) represents the compressive force acting on the coronary vessels due to the contraction and relaxation of the left ventricles. It is proportional to the left ventricular (LV) pressure by the constant *K*. At time *t*, the pressure *P*(*t*) is related to the flow rate *Q* and the *P_im_*(*t*) by the following relationship:
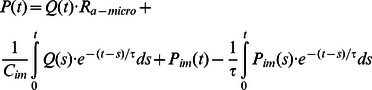
(1)Where *τ* is the time constant and is defined as follow:
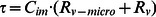
(2) With this relationship, the lumped parameter model can be coupled to the outlet boundary of the computational domain Г as follows:
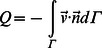
(3)

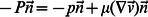
(4) Where the main quantities from the lumped parameter model (*Q, P*) are coupled with ones from the three-dimensional model (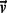,*p*) [13]–[14].

The inlet pressure and the corresponding LV pressure for rest and exercise conditions can be obtained from Kim *et al.* 's model and the profiles were shown in Figure 2. At rest, the heart rate was 60 beats/min and the maximum systole inlet pressure was 123 mmHg. During exercise, the heart rate increased to 120 beats/min and the maximum systole inlet pressure slightly increased to 133 mmHg. The no slip condition was applied on the vessel wall.

### Parameter Values

Under resting condition, the parameter values for non-CT models are: *R_a-micro_*  =  148×10^3^ dynes s/cm^5^, *R_v-micro_* + *R_v_* = 65×10^3^ dynes s/cm^5^ and *C_im_* = 4.16×10^−6^ cm^5^/dynes [10]. While for tortuous coronary models, we decreased the resistance of the downstream models until we obtained the same mean flow rate with the corresponding non-CT case. With this approach, the auto-regulation effect of the coronary arteries was modeled. Under exercise condition, two experiments were designed to investigate the impact of CT. First, the maximal dilatation condition was modeled for two cases. The maximal dilatation condition means the distal vessels are in a state of hyperemia and the resistances are reduced to the minimum. Under this extreme condition, the blood supply reaches the maximum and the results will directly show the impact of tortuosity on the maximal flow rate. The maximal dilatation condition employed here was based on the work of Roberto Burattini *et al.* and was first used in CFD simulation by Kim *et al.* [10], [15]. The parameter values are: *R_a-micro_* = 24×10^3^ dynes s/cm^5^, *R_v-micro_* + *R_v_* = 19×10^3^ dynes s/cm^5^ and *C_im_* = 15.9×10^−6^ cm^5^/dynes [10]. Second, under the maximal dilatation condition, we assumed that the auto-regulation effect still had the ability to maintain the flow rate. Thus we decreased the resistance of the downstream vascular beds for CT models to get the same mean flow rate with the corresponding non-CT case. Indeed, the resistances cannot be reduced anymore in the maximal dilatation condition. However, by assuming that the auto-regulation effect is still working, we can get the changes in distal resistances that would be required to maintain blood perfusion, in order to directly show the impact of the CT on the blood supply regulation. In the lumped parameter model, the parameter *K* was adjusted to give physiologically realistic coronary flow and pressure waveforms and was set to be 0.8 for all cases.

### Computational Method

The numerical simulation was achieved by using the CFD software ANSYS FLUENT (ANSYS Inc.). ANSYS FLUENT is finite-volume-based software for fluid mechanics computations. Blood was modeled as an incompressible Newtonian fluid with the dynamic viscosity *μ* and the density *ρ*, respectively, set to 4 cP and 1070 kg/m^3^. Mesh independence was judged by comparing both the computed velocities and the computed pressure. For each case, the computed velocity profiles for the finally chosen mesh differed by 2% from those for doubled mesh resolution. Mesh independence for the pressure was checked and the difference of the pressure at outlet between the current selected mesh and doubled resolution mesh was less than 1%. The solutions were run until the pressure fields at the outlet did not change more than 1% compared to the solutions at the same phase in the previous cardiac cycle.

## Results

### Flow Rate and Downstream Perfusion Pressure

**Figure 3:**
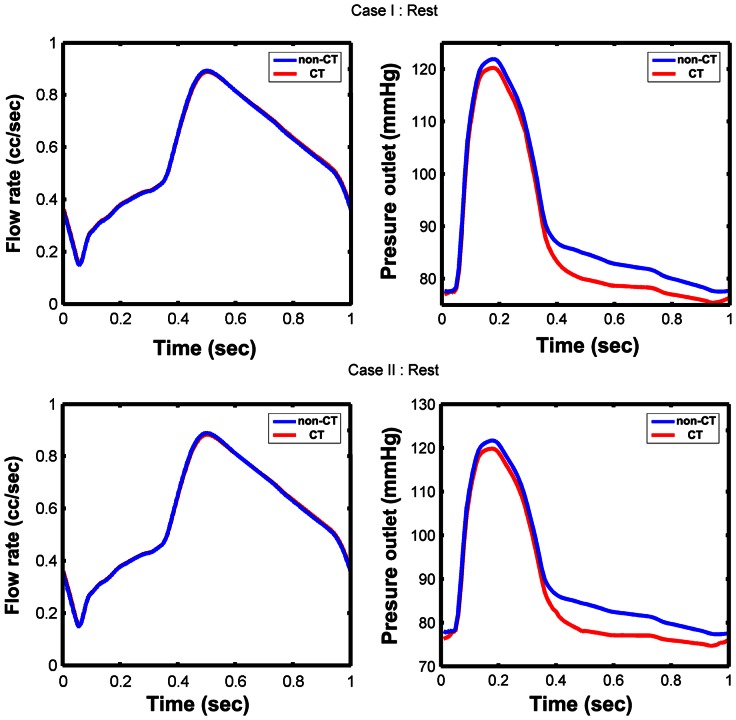
The flow rate and downstream perfusion pressure for two cases under resting condition.

Under resting condition, the mean flow rates for non-CT cases were: 0.57 cc/sec for Case I and 0.56 cc/sec for Case II. To get the same flow rate, the parameter values for CT models were reduced to *R_a-micro_* = 140×10^3^ dynes s/cm^5^, *R_v-micro_* + *R_v_* = 61×10^3^ dynes s/cm^5^ for Case I and *R_a-micro_* = 137×10^3^ dynes s/cm^5^, *R_v-micro_* + *R_v_* = 59×10^3^ dynes s/cm^5^ for Case II. The flow rate and downstream perfusion pressure under resting condition were shown in Figure 3. For Case I, the mean downstream perfusion pressure was 90.7 mmHg for the non-CT model and 87.8 mmHg for CT model. For Case II, the mean downstream perfusion pressure was 90.3 mmHg for non-CT model and 86.5 mmHg for CT model. The results clearly showed that the downstream perfusion pressure would decrease due to tortuosity, especially in diastole.

**Figure 4:**
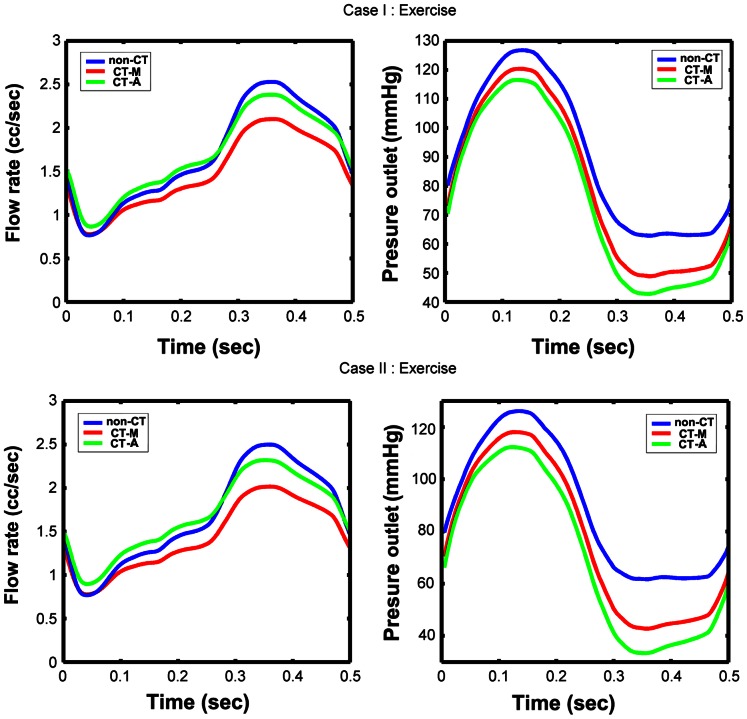
The flow rate and downstream perfusion pressure for two cases during exercise. non-CT: non-CT models during exercise condition; CT-M: CT models during the maximal dilatation condition; CT-A: CT models with the auto-regulation effect during exercise condition.

During exercise, the maximal dilatation for the downstream coronary vasculatures was modeled. The flow rate and downstream perfusion pressure were shown in Figure 4. From the results we find that both the flow rate and the downstream perfusion pressure decreased due to tortuosity. For Case I, the mean flow rate was 1.7 cc/sec for non-CT model; while it decreased to 1.48 cc/sec for CT model. The same phenomenon was observed in Case II, the mean flow rate was 1.68 cc/sec for non-CT model and it decreased to 1.43 cc/sec for CT model. The mean downstream perfusion pressure was 89.7 mmHg for Case I non-CT model, 80.6 mmHg for Case I CT model, 88.8 mmHg for Case II non-CT model and 77.0 mmHg for Case II CT model. In diastole, the downstream perfusion pressure of CT models was significant lower than that of non-CT models for both cases. A maximum of 19.0 mmHg decrease was observed in diastole due to tortuosity. By assuming that the auto-regulation effect still has the ability to maintain the flow rate, the changes in distal resistances that would be required to maintain perfusion for CT models were obtained. The parameter values for the CT models were reduced to *R_a-micro_* = 18.5×10^3^ dynes s/cm^5^, *R_v-micro_* + *R_v_* = 14.5×10^3^ dynes s/cm^5^ for Case I and *R_a-micro_* = 17.5×10^3^ dynes s/cm^5^, *R_v-micro_* + *R_v_* = 13.5×10^3^ dynes s/cm^5^ for Case II.

### Velocity Field

**Figure 5:**
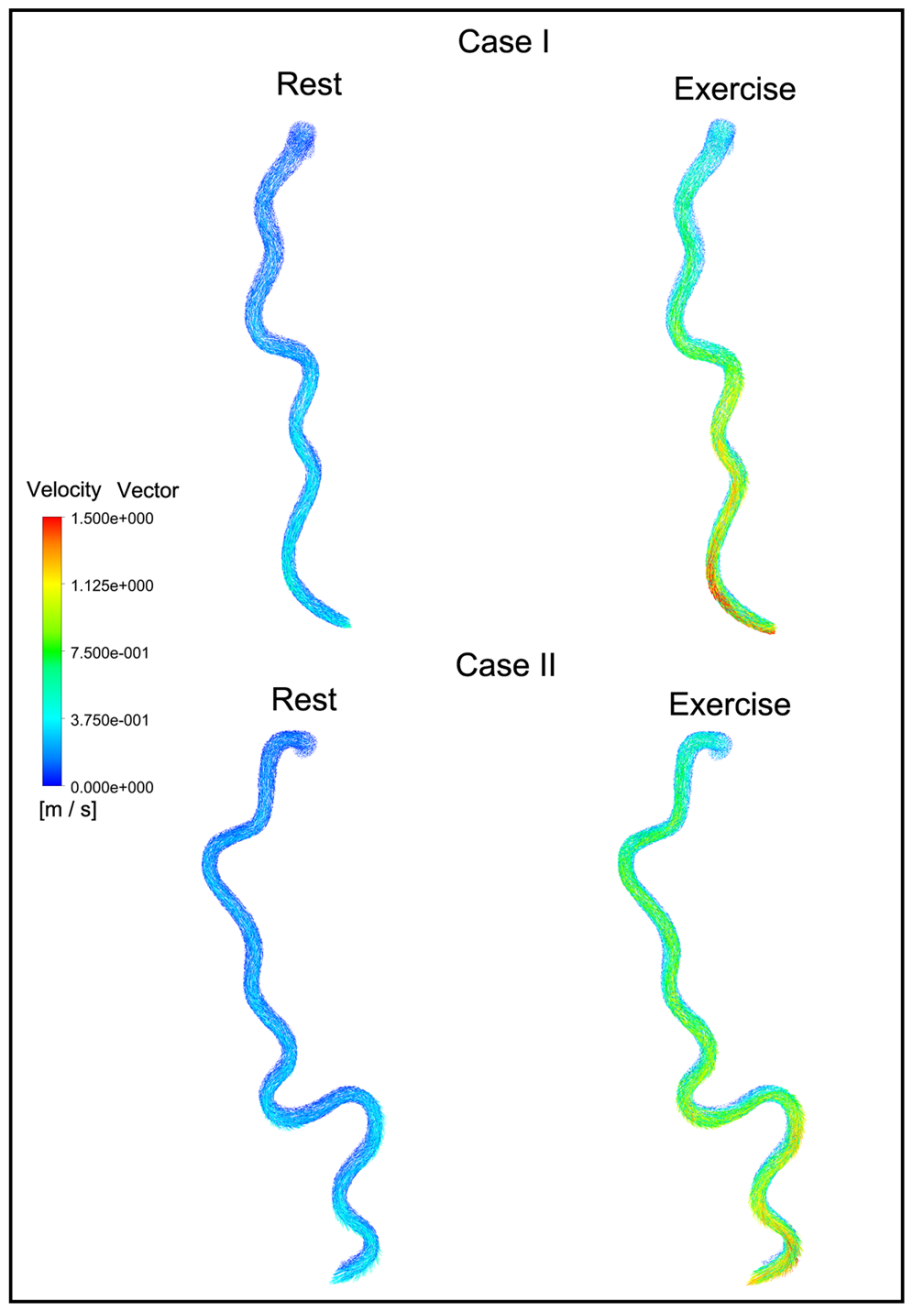
The velocity vectors at the maximal flow rate frames for CT models during rest and exercise conditions. Time frames were: t = 0.47 s for rest condition and t = 0.35 s for exercise condition (the maximal dilatation condition).

Velocity field was presented at the maximal flow rate frames for CT models during rest and exercise conditions. Time frames were: *t* = 0.47 s for rest condition and *t* = 0.35 s for exercise condition (the maximal dilatation condition). The velocity vectors were shown in Figure 5. From the results, we find that the fastest particles were pressed outwards at the bend sections by the centrifugal effect; the original symmetrical velocity profile skewed toward the outer wall of the bend. This phenomenon was more pronounced during exercise, as compared to rest condition.

### Driving Pressure

**Figure 6:**
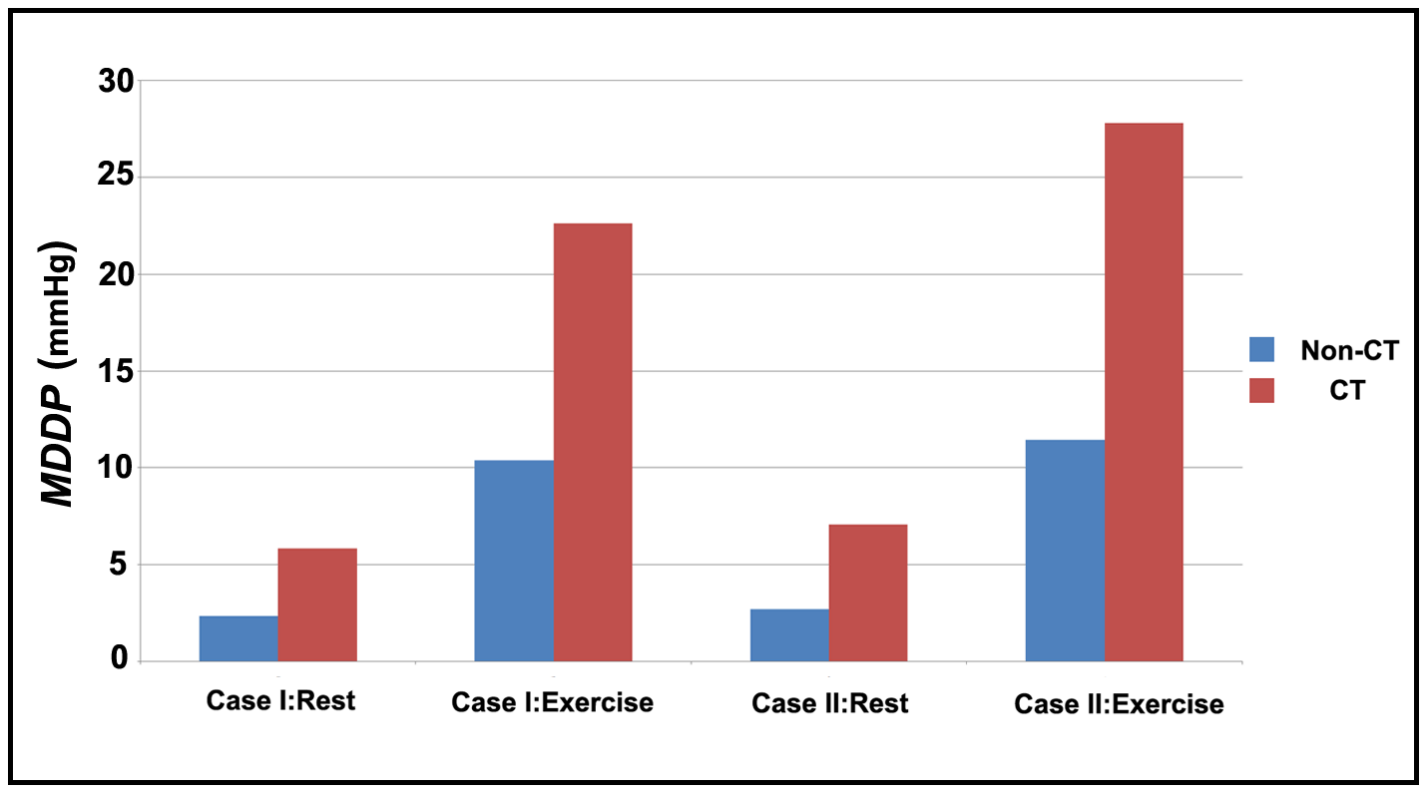
The MDDP for all simulated cases at rest and during exercise.

**Figure 7:**
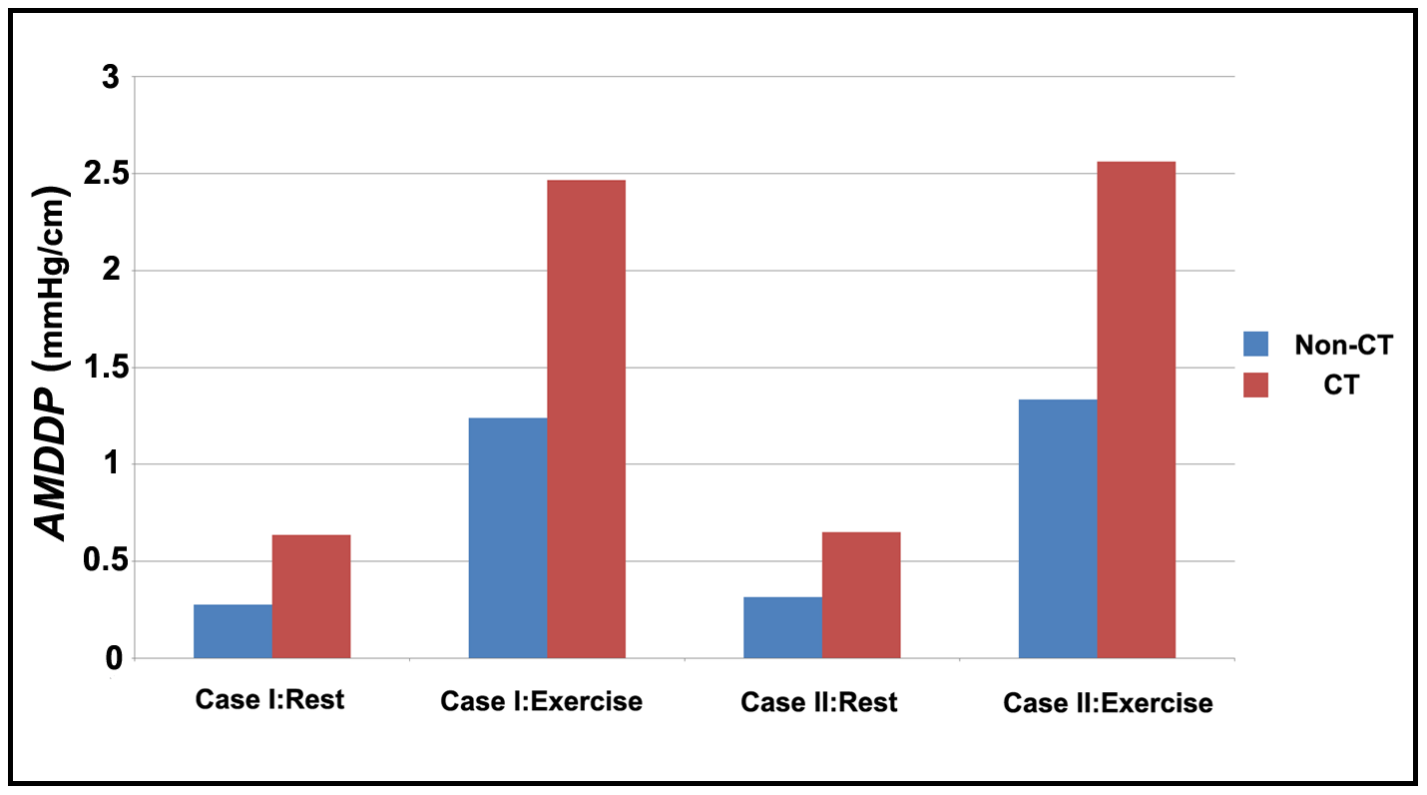
The AMDDP for all simulated cases at rest and during exercise.

Pressure gradient required to drive the blood through a given arterial segment is an important hemodynamic parameter. Extravascular compression during the systole markedly affects the coronary flow and most of the coronary flow occurs during the diastole. Therefore the mean diastole driving pressure (*MDDP*) (the pressure drop between the inlet and the outlet) for rest and exercise conditions (the maximal dilatation condition) were investigated and shown in Figure 6. The curve lengths between CT models and non-CT models are not equal, thus the averaged mean diastole driving pressure (*AMDDP*) (the ratio of *MDDP* over the curve length) was also given in Figure 7. Results indicated that tortuosity lead to an increase in both *MDDP* and *AMDDP*. These increases became notable during exercise. Compared to non-CT models, the *MDDP* increased 3.5 mmHg at rest and 12.2 mmHg during exercise for Case I, and it increased 4.4 mmHg at rest and 16.3 mmHg during exercise for Case II. For CT models, the *AMDDP* also increased more as compared to non-CT models. These increases were 0.36 mmHg/cm for Case I under resting condition, 1.23 mmHg/cm for Case I during exercise condition, 0.34 mmHg/cm for Case II under resting condition and 1.22 mmHg/cm for Case II during exercise condition.

### Resistance

**Figure 8:**
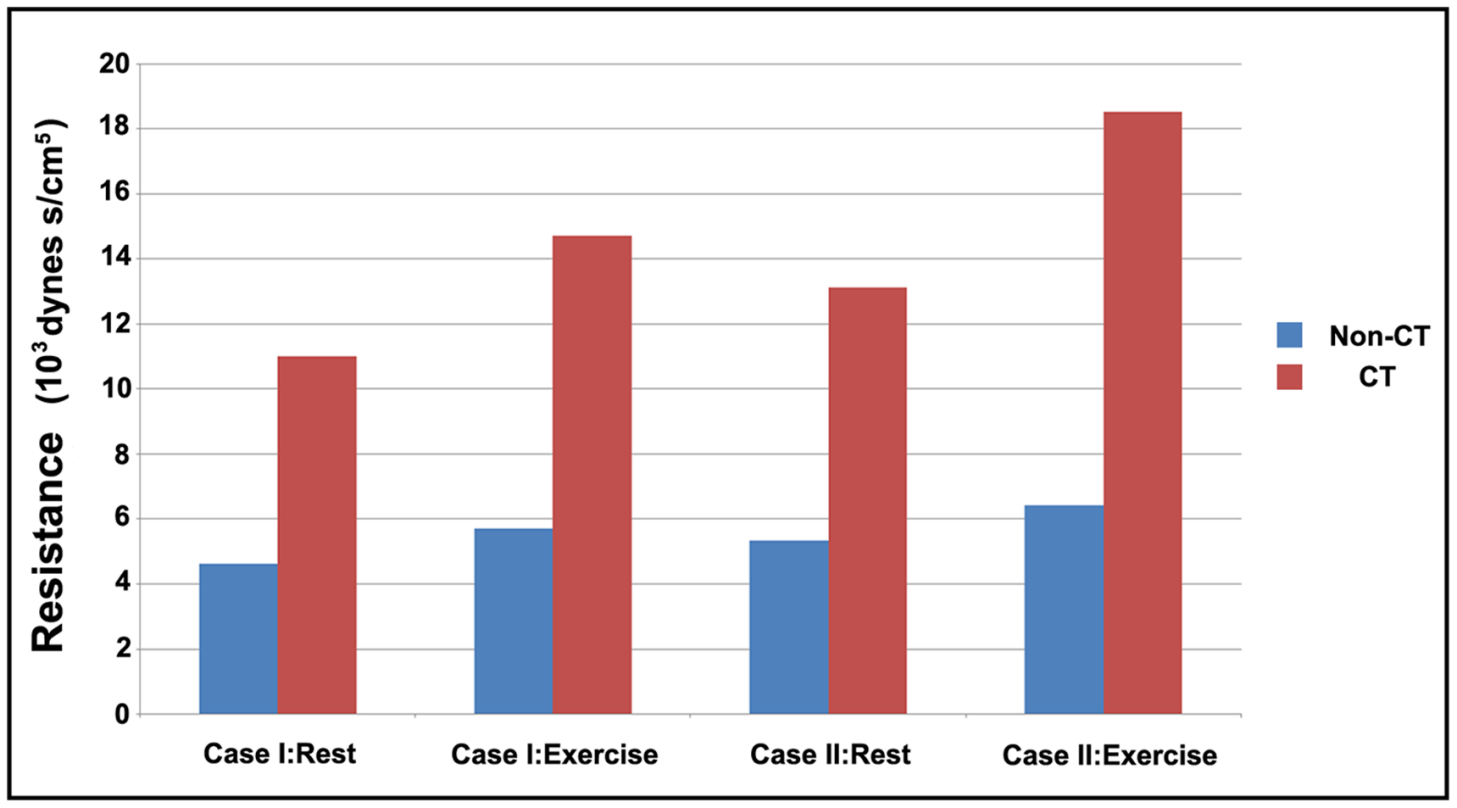
The resistances for all simulated cases at rest and during exercise.

Resistance is defined by the ratio of mean driving pressure over the flow rate. The resistances for different models under rest and exercise (the maximal dilatation condition) conditions were shown in Figure 8. Exercise would increase the flow rate, and further make the resistance of the simulated branches increased. For non-CT models, the resistance increased from 4.61×10^3^ dynes s/cm^5^ to 5.33×10^3^ dynes s/cm^5^ for Case I and increased from 5.7×10^3^ dynes s/cm^5^ to 6.41×10^3^ dynes s/cm^5^ for Case II. For CT models, the resistance increased from 11.01×10^3^ dynes s/cm^5^ to 14.71×10^3^ dynes s/cm^5^ for Case I and increased from 13.11×10^3^ dynes s/cm^5^ to 18.51×10^3^ dynes s/cm^5^ for Case II. It was obviously that the resistance of CT models was larger than that of non-CT models, especially during exercise.

To clarify the relative contributions of lengthen and curvature effects to the increase in flow resistance with tortuosity, another non-tortuous model was constructed for each case with the curve length and the diameter were as same as those of the CT model. The curvatures of these non-CT models were set to be the mean curvature of the corresponding CT models.

The resistances of these non-tortuous models are: 5.79×10^3^ dynes s/cm^5^ for Case I under rest condition (with a mean flow rate of 0.57 cc/sec); 7.01×10^3^ dynes s/cm^5^ for Case I during exercise (with a mean flow rate of 1.63 cc/sec); 7.63×10^3^ dynes s/cm^5^ for Case II under rest condition (with a mean flow rate of 0.56 cc/sec) and 9.22×10^3^ dynes s/cm^5^ for Case II during exercise (with a mean flow rate of 1.58 cc/sec). These results indicated that the increase in flow resistance with the lengthen effect only takes a small part of the total increase in flow resistance with tortuosity.

## Discussion

Tortuosity may affect the coronary blood flow and cause a reduction in coronary pressure [7]. Whether this reduction is significant and can lead to myocardial ischemia is still unknown. To determine the impact of tortuosity on coronary blood supply, we performed a quantitative analysis of the coronary flow in two patient-specified tortuous LAD models during rest and exercise conditions. The lumped parameter model was coupled to the outlet of the three-dimensional coronary models to represent the absent downstream vasculatures. For each case, a corresponding non-CT model was constructed to add a comparison between the CT and non-CT models. In this work, the investigated parameters comprised the flow rate, the downstream perfusion pressure, the *MDDP*, the *AMDDP* and the resistances.

When blood flows through arteries, the pressure drops due to the energy loss [16]. At rest, the pressure drop was small as compared to the coronary pressure for all cases. However, during exercise, the pressure drop for the CT cases would increase to a significant level. When the fluid flows through a tortuous tube, the curvature of the tube may generate centrifugal forces that induce secondary flows causing variations in the friction factor [17]–[18]. There are two causes for the energy loss when blood flows through tortuous arteries [3]. One is the friction through the shear stress and the other is the centrifugal effect. Compared to non-CT models, CT models have some bend sections and are longer than non-CT models. Thus, extra energy would be lost due to the bend section effect (the centrifugal effect) and the lengthen effect (the friction) as blood flowing through CT models. Compared to non-CT models, the *MDDP* for CT models increased about 122% for Case I and 141% for Case II. At the same time, the *AMDDP* for CT models increased about 99% for Case I and 90% for Case II as compared to non-CT models. These results indicated the two important geometry changes (lengthen and bend sections) caused by tortuosity both contribute to the extra energy loss caused by CT, and the extra energy loss caused by the bend section effect (the centrifugal effect) took more part of the total extra energy loss during exercise. Comparing the resistances between the CT models and the non-tortuous models (the non-tortuous models with the same curve length as of the CT models), the bend section effect played a more important role in determining the flow resistance.

From the results above, the blood flow rate of coronary arteries would reduce notably due to tortuosity during exercise (the maximal dilatation condition). While under resting condition, the influence of tortuosity on the blood flow rate is negligible. As compared to the resistance of the downstream vascular bed, the resistances of the simulated branches were much smaller under resting condition. At this time, although the downstream perfusion pressure reduced due to tortuosity, the auto-regulation effect could be easily achieved by decreasing the resistance of the downstream vascular bed. In this simulation work, we decreased the resistance of the downstream vascular bed for CT cases to model the auto-regulation effect under resting condition. Results showed that the mean flow rate could be maintained by decreasing less than 8% of the downstream vascular bed's resistance under resting condition. While during exercise (the maximal dilatation condition), to maintain the mean flow rate, the resistances of the downstream vascular bed would be decreased more than 23%. Under exercise condition, the resistance of the downstream vascular bed decreased to about 20% of basal resting values. In this situation, the resistance of the simulated branches played an important role in determining the flow rate. Under the maximal dilatation condition, the total coronary resistance increased 20.3% and 25.5% in case I and case II respectively. These increases in the resistance lead to a decrease in the flow rate for the CT cases as compared to the non-CT models. The mean flow rate decreased by 12.9% for Case I and 14.9% for Case II due to tortuosity. Under the maximal dilatation condition, the distal resistances were reduced to the minimum. However, the distal resistances for CT models still have to reduce 23% for Case I and 28% for Case II to maintain the perfusion. These results implied that patients with CT may lack the ability to adjust distal resistance sufficiently to compensate for the extra resistances generated by tortuosity during exercise.

As in all vascular beds, it is small arteries and arterioles in the microcirculation that are the primary sites of vascular resistance [8]. During exercise, the effective coronary perfusion pressure increases by no more than 20–30% [8]. While the coronary blood flow increases 3 to 5-fold by a decrease in the coronary vascular resistance. However, in the tortuous coronary arteries, exercise would increase the flow rate, and further make the resistance of the tortuous sections increased. On the one hand, the resistance of small arteries reduces to 20–30% of basal resting values [8]. On the other hand, the resistance of the tortuous section increases to nearly 300% as compared to non-CT cases. Much more parts of the total coronary perfusion pressure will be used to drive blood to travel through the tortuous section. From the results above, these changes will cause the regulation of blood flow ineffective and the demand of the myocardial blood supply may not be met during exercise.

The main focus of this study was to investigate the impact of tortuosity on the coronary blood supply. Under resting condition, the mean coronary flow rate could be easily maintained by the auto-regulation effect, while during exercise, a notable reduction in the maximal mean coronary flow rate could be observed due to tortuosity. These results are consistent with the clinical phenomenon. During exercise, CT may cause an insufficient perfusion pressure and lead to ischemia. While at rest, through the auto-regulation effect, the blood supply of CT patients can easily be maintained. This explains why patients with the severe CT often suffer the exercise-induced chest pain and typically disappear under resting condition.

We have to clarify that this study has some limitations. Due to the low resolution of the computerized tomography images, some small branches of the LAD models were removed from the reconstructed model. In this work, the elasticity of the vessel was ignored. The fluid-structure interaction analysis can be applied to include the property of the vessel elasticity [18]. However, it has been reported that the coronary artery deformation in the radial direction is small [19]. Previous studies also have shown that the wall elasticity has little impact on the flow field [20]–[21]. So the elasticity of the vessel was ignored. The motion of the heart during the cardiac cycle was not considered. The arteries were fixed to the surface of the heart through the simulation. In reality, the coronary vascular bed has repetitive flexion and relaxation during each cardiac cycle. The dynamic motion of coronary arteries is large and cannot be modeled using this approach. However, it has been reported that the dynamic motion of coronary arteries has limited influence on the flow field and the pressure [19], [22]–[23].

To the best of our knowledge, this is the first three-dimensional computational patient-special study to investigate the impact of CT on the coronary blood supply. Two patient-specific models and the responding non-CT models were employed to perform the CFD simulation. The lumped parameter model was coupled to the outlet of the simulated branches to represent the absent downstream vasculatures. Results indicated that during resting conditions, the mean flow rate could be maintained by decreasing less than 8% of the downstream vascular bed's resistance for tortuous models; while during exercise, the maximal coronary blood supply would reduce 12.9% for Case I and 14.9% for Case II due to tortuosity. Assuming that the flow rate can be maintained by auto-regulation effect, the distal resistances for CT models still have to reduce 23% for Case I and 28% for Case II to maintain the perfusion. The main reasons for these results were: (i) the resistance of the downstream vascular bed was much larger than that of the simulated LAD branches during resting condition and the flow rate was mainly determined by the resistance of the downstream vascular bed; (ii) the resistance of the downstream vascular bed decreased much during exercise and thus the flow rate became sensitive to the resistance of the simulated branches during exercise; (iii) CT would increase the resistance of the tortuous section, especially during exercise condition. Coronary tortuosity has minor influence on coronary blood supply at rest; while during exercise, patients with CT may lack the ability to adjust distal resistance sufficiently to compensate for the extra resistances generated by tortuosity and this may further lead to an ineffective regulation of the blood flow.
